# Successful establishment of primary small airway cell cultures in human lung transplantation

**DOI:** 10.1186/1465-9921-10-99

**Published:** 2009-10-26

**Authors:** Balarka Banerjee, Anthony Kicic, Michael Musk, Erika N Sutanto, Stephen M Stick, Daniel C Chambers

**Affiliations:** 1 School of Paediatrics and Child Health, University of Western Australia, Nedlands, 6009, Western Australia, Australia; 2 School of Medicine and Dentistry, University of Western Australia, Nedlands, 6009, Western Australia, Australia; 3 Western Australia Lung Transplant Program, Royal Perth Hospital, Perth, 6000, Western Australia, Australia; 4 Department of Respiratory Medicine, Princess Margaret Hospital for Children, Perth, 6001, Western Australia, Australia; 5 Telethon Institute for Child Health Research, Subiaco, 6008, Western Australia, Australia; 6 Queensland Centre for Pulmonary Transplantation and Vascular Disease, The Prince Charles Hospital, Brisbane, 4032, Queensland, Australia

## Abstract

**Background:**

The study of small airway diseases such as post-transplant bronchiolitis obliterans syndrome (BOS) is hampered by the difficulty in assessing peripheral airway function either physiologically or directly. Our aims were to develop robust methods for sampling small airway epithelial cells (SAEC) and to establish submerged SAEC cultures for downstream experimentation.

**Methods:**

SAEC were obtained at 62 post-transplant bronchoscopies in 26 patients using radiologically guided bronchial brushings. Submerged cell cultures were established and SAEC lineage was confirmed using expression of clara cell secretory protein (CCSP).

**Results:**

The cell yield for SAEC (0.956 ± 0.063 × 10^6^) was lower than for large airway cells (1.306 ± 0.077 × 10^6^) but did not significantly impact on the culture establishment rate (79.0 ± 5.2% vs. 83.8 ± 4.7% p = 0.49). The presence of BOS significantly compromised culture success (independent of cell yield) for SAEC (odds ratio (95%CI) 0.067 (0.01-0.40)) but not LAEC (0.3 (0.05-1.9)). Established cultures were successfully passaged and expanded.

**Conclusion:**

Primary SAEC can be successfully obtained from human lung transplant recipients and maintained in culture for downstream experimentation. This technique will facilitate the development of primary *in vitro *models for BOS and other diseases with a small airway component such as asthma, cystic fibrosis and COPD.

## Background

Although lung transplantation is a well-accepted therapeutic option for selected patients with advanced lung disease, long-term survival is limited largely by progressive and treatment refractory airflow limitation manifest clinically as bronchiolitis obliterans syndrome (BOS) [1]. The predominant histopathologic finding in patients with BOS is of fibro-proliferative small airway obliteration (obliterative bronchiolitis (OB)). Unfortunately, there has been no substantial improvement in the reported incidence of BOS over the last twenty years, despite improvements in immunosuppression, surgical techniques, and patient management [2,3]. Recognition of the central role of OB in limiting post-transplant survival has led to a large body of research aimed it improving management [4-7]. However, all *in vitro *human studies have used large airway epithelial cells (LAEC) despite OB being predominantly a small airway disease. The aim of this study was to develop methodology for the successful sampling and culture of small airway epithelial cells (SAEC) obtained from lung transplant patients at routine post-transplant bronchoscopy. The described techniques will provide a more relevant *in vitro *human cell based model to study the pathogenesis of OB.

## Methods

### Reagents

Foetal Calf Serum (FCS), RPMI-1640 media, penicillin G, streptomycin sulphate, amphotericin B and L-glutamine were purchased from Invitrogen (Melbourne, Australia). Insulin, bovine serum albumin (BSA), hydrocortisone, recombinant human epidermal growth factor (EGF), epinephrine hydrochloride, triiodothyronine, retinoic acid, trypsin and gentamycin were obtained from Sigma (St. Louis, USA). Bronchial epithelium basal medium (BEBM) was purchased from LONZA™ (Basel, Switzerland). Ultroser G was supplied from Ciphergen (Cergy-Saint-Christophe, France). Collagen S (type I) as well as fibronectin were purchased from Roche (Dee Why, Australia). All tissue culture plastic ware was obtained from Sarstedt (Mawson Lakes, Australia).

### Patients

A total of 62 bronchoscopies were performed in 26 patients (11 female; aged 18 to 64 years (median 51 years); 4 BOS). Patient demographics are summarized in Table 1. BOS was diagnosed and graded according to international guidelines [2]. The study was approved by the Royal Perth Hospital Human Research and Ethics Committee.

**Table 1:** Demographics of patients sampled

Patient No.	Sex	Age	Type of Transplant	Reason for transplant	Months post transplant at brushing
**1**	f	18	Bilateral	Pulmonary capillary haemangiomatosis	4,5,13
**2**	m	50	Single	Usual interstitial pneumonia	15
**3**	m	46	Heart-lung	Congenital heart disease	72
4	f	55	Single	Usual interstitial pneumonia	11,16
**5**	m	40	Bilateral	Usual interstitial pneumonia	8,10,16,19,21
**6**	m	61	Bilateral	COPD	5,9
**7**	m	60	Bilateral	COPD	3,4,7
**8**	f	25	Bilateral	Cystic fibrosis	1,2,3,6
**9**	m	58	Bilateral	Bronchiectasis	14,27
**10**	f	55	Single	Usual interstitial pneumonia	10, 13, 19
**11**	f	58	Single	Usual interstitial pneumonia	22
**12**	m	59	Single	Usual interstitial pneumonia	7, 10,13
**13**	f	43	Bilateral	Congenital heart disease	2,3,4
**14**	m	44	Bilateral	Cystic fibrosis	3,6,7,10,11
**15**	m	40	Heart-lung	Congenital heart disease	118, 129
**16**	m	51	Bilateral	Sarcoidosis	6,7,10,18
**17**	f	58	Bilateral	COPD	39
**18**	m	51	Bilateral	Cystic fibrosis	1,2,2,4,10
**19**	m	64	Bilateral	COPD	108,109
**20**	m	49	Bilateral	COPD	10
**21**	f	61	Single	COPD	3,6
**22**	f	54	Single	Usual interstitial pneumonia	4,6
**23**	m	59	Bilateral	Cystic fibrosis	4,5
**24**	m	54	Single	Usual interstitial pneumonia	1
**25**	f	25	Heart-lung	Congenital heart disease	2
**26**	f	27	Bilateral	Cystic fibrosis	6

*COPD - Chronic Obstructive Pulmonary Disease

### Bronchoscopy procedure

**Figure 1:**
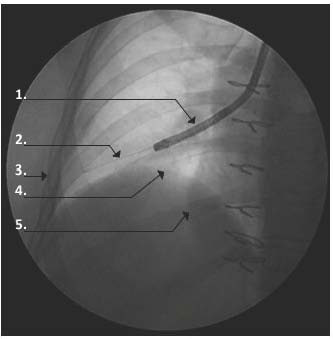
**Brushing of small airways under radiological guidance**. A nylon cytology brush is guided down the working channel of a standard bronchoscope and then extended to reach 2-3 cm from the pleural surface. **1**. Bronchoscope; **2**. Cytology brush; **3**. Pleural surface; **4**. Diaphragm; **5**. Right heart border.

Human airway epithelial cells (AEC) were collected using a bronchial brush during routine surveillance and diagnostic post-transplant bronchoscopies. Bronchoscopy was conducted under general anaesthesia with a laryngeal mask or endotracheal tube. The bronchoscope(Olympus^® ^Evis EXERA II) was wedged in a suitable lateral segment of the right or left lower lobe. Prior to the acquisition of transbronchial biopsies, the sheathed nylon cytology brush (10 mm, 2 mm outer diameter, Olympus BC-25105, Waverley, Australia) was passed down the working channel of the bronchoscope and then unsheathed under radiological guidance with the brush tip lying 2-3 cm from the pleural surface. Small airway brushings (2-3 brushings) were collected from this area (Fig. 1).

Large airways brushings (*2-3) were obtained from segmental bronchi in the standard way. In both cases, brushings were collected into a tube containing 2 ml of RPMI on ice and the brush tip was also cut off and collected after the final brushing. After the completion of brushings, 20% (v/v) FCS was added to the tubes and processed immediately. Tubes and brushes were kept separate for large and small airways to prevent cross contamination. The lung apex was screened to exclude a pneumothorax as part of routine care following a transbronchial biopsy.

### Establishment of cultures

During processing, the cells were fractionated for RNA archiving, cytospins and cell culture as previously described [8]. Successful culture establishment was assessed as previously described [8] and cultures were passaged at 90% confluence. Additionally, a human bronchial epithelial cell line (16HBE14o^-^; provided by Dieter Gruenert, University of California San Francisco, USA) was also utilised and maintained as previously described [8].

### Growth media and culture conditions

Primary SAEC and LAEC were maintained in Bronchial Epithelial Basal Media (BEBM) (Lonza™) supplemented with 2% (v/v) Ultroser G, 50 μg/mL bovine pituitary extract, 0.5 μg/mL hydrocortisone, 5 ng/mL human epidermal growth factor, 0.5 μg/mL epinephrine, 6.5 ng/mL triiodothyronine, 5 μg/mL insulin, 1 ng/mL retinoic acid, 10 μg/mL transferrin, and 0.001% gentamycin (v/v). 16HBE14o^- ^cells were maintained in Dubelco's Minimum Essential Media (DMEM) (Invitrogen (Melbourne, Australia)), FCS (10%, v/v), penicillin (100 U/ml), streptomycin (100 μg/ml) and amphotericin B (2.5 μg/ml). All cell cultures were grown in a NUAIRE (Plymouth, USA) incubator at 37°C in an atmosphere of 5% CO_2_/95% air under strict aseptic conditions.

### Epithelial lineage verification

Cells from the cultures before the first passage (p0) and after the second passage (p2) of both SAEC and LAEC were cytospun onto glass slides and epithelial lineage verified by immunocytochemistry (ICC) as previously described [8]. First passage (p0) and p2 were specifically chosen because most experiments were conducted at p2 and cells were generally not propagated beyond that. Briefly, cytospins were incubated with primary antibodies specific for mesenchymal (Vimentin 1:250) (Santa Cruz Biotechnology Inc., Santa Cruz, USA), endothelial (von Willebrand factor 1:500) (Santa Cruz Biotechnology Inc., Santa Cruz, USA), macrophage (CD68 1:500) (DAKO Corp, Carpinteria, USA), dendritic (CD1a 1:250) (Santa Cruz Biotechnology Inc., Santa Cruz, USA), and epithelial lineages (AE1-AE3 1:250) (DAKO Corp, Carpinteria, USA) for 24 hours at 4°C followed by fluorescently conjugated secondary antibodies for a similar period. Secondary antibodies included; anti-mouse FITC conjugate (1:100), anti-goat FITC conjugate (1:100) and anti-rabbit FITC conjugate (all Sigma, St. Louis, USA). The slides were observed under a fluorescent microscope (Leica Microsystem Pty. Ltd., Wetzlar, Germany) for staining. AE1-AE3 was chosen as the positive marker for epithelial cells since it is a mixture of clones AE1 and AE3. AE1 detects high molecular weight keratins 10, 14, 15, and 16 as well as low molecular weight cytokeratin-19. Clone AE3 detects the high molecular weight cytokeratins 1, 2, 3, 4, 5, and 6, and the low molecular weight cytokeratins 7 and 8. By combining the two reagents a broad spectrum of reactivity is achieved.

### Small airway epithelial lineage verification

When cells reached 90% confluence in culture, they were trypsinised, collected and resuspended in 1 ml of RPMI. The cell suspension was then fractionated and a 350 μl aliquot was centrifuged and pelleted cells stored in RLT buffer (QIAGEN, Hamburg, Germany). RNA was extracted using QIAGEN RNA Easy Mini Kit. The remainder of the cell suspension was seeded into pre-coated flasks to continue propagation of the culture. Lineage was verified by analysing mRNA from a representative group of cultures chosen at random from patients who were not diagnosed with BOS. Quantitative polymerase chain reaction (qPCR) was used to assess expression of the Clara Cell Secretory Protein (CCSP), which is uniquely expressed by small airway epithelial cells, and surfactant protein B (SP-B), which is commonly expressed by non-ciliated bronchial epithelial cells [9] and type II alveolar cells [10]. Lineage verification was carried out on mRNA from cultures at p0 and p2. Gene expression was analyzed using two-step reverse transcription polymerase chain reaction (RT-PCR) and cDNA synthesized using hexanucleotide primers and Multiscribe™ Reverse Transcriptase (Applied Biosystems, Foster City, USA) in a final reaction volume of 20 μL containing 1 × RT buffer (Promega Madison, USA), 5.5 mM MgCl_2_, 0.5 mM of each of the dNTPs, 2.5 μM random hexamers, 0.4 U RNase inhibitor, 1.25 U Multiscribe (Applied Biosystems, Foster City, USA) reverse transcriptase and 200 ng RNA. All reactions were performed under the following conditions: initial primer incubation step at 25°C for 10 minutes followed by RT incubation at 48°C for 1 hour and ended by reverse transcriptase inactivation at 95°C for 5 minutes. The cDNA was then used in a final PCR reaction volume of 25 μL containing 1× Sybr Green PCR master mix (Applied Biosystems, Foster City, USA), 0.5 μM each of forward and reverse primers and 5 μL of cDNA (1:5). The conditions for the PCR include initial incubation at 50°C for 2 minutes, AmpliTaq Gold activation at 95°C for 10 minutes followed by 40 cycles of 15 seconds at 95°C and 1 minute at 60°C. The sequences of the primers used included;*CCSP*; forward; '5AAACCCTCCTCATGGACACAC3' and reverse '3GACGGTACGAAACTCAGGT5', *SP-B*; forward; '5TCACACACAGGATCTCTCCG3' and reverse 3'AGGTCGTGGTAGGTGTGGAG5', *18S*; forward; '5TAACCCGTTGAACCCCATTC3' and reverse '3TCCAATCGGTAGTAGCGACG5'.

Quantitative PCR was performed using the ABI Prism 7700 Sequence Detection System (Perkin-Elmer, USA) and signals were analyzed by the ABI Prism Sequence Detection System software version 1.9. Expression of CCSP and SP-B was quantified relative to the expression of 18S.

### Statistics

All results were tested for population normality and homogeneity of variance and are presented as mean ± SEM unless otherwise specified. Comparisons were made using odds ratios for dichotomous variables and Student's t-test for continuous variables. p values < 0.05 were considered to be significant.

## Results

**Figure 2:**
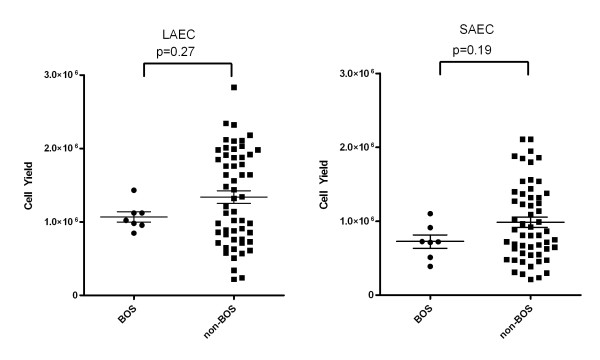
**Cell yield from brushing LAEC and SAEC of BOS v. non-BOS patients**. No significant difference was noted between the yield in SAEC and LAEC from BOS v. non-BOS patients.

Brushings were successfully obtained from both the small and large airways of the transplanted lung at all 62 bronchoscopies. The brushing method was well tolerated by all patients with no significant bleeding, pneumothorax or other adverse events being observed. The mean cell yield from the allograft was significantly higher for LAEC (1.306 ± 0.077 × 10^6^) than for SAEC (0.956 ± 0.063 × 10^6^; p < 0.01). No significant difference in yield was noted between BOS and non-BOS patients (Fig. 2).

### Cell culture establishment

**Figure 3:**
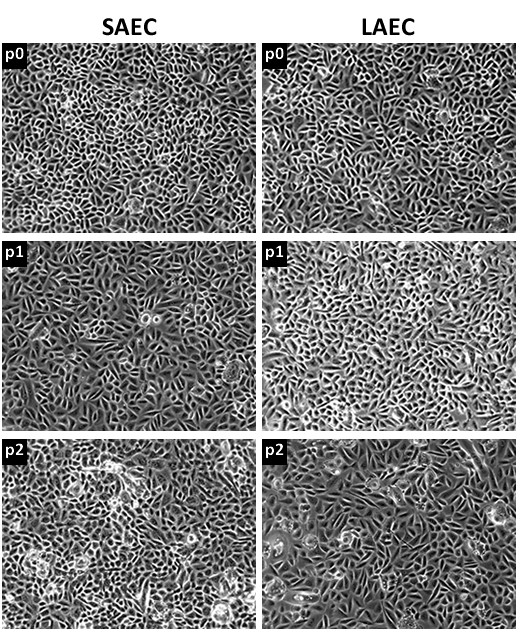
**Morphology of epithelial cells is maintained over passage**. Phase contrast micrographs showing no morphological variation in bronchial epithelial cells cultured from small airway (SAEC) and large airway (LAEC) of a lung allograft, obtained during routine bronchoscopy. All cells exhibited a cobblestone morphology which was maintained over two consecutive passages (p0 to p2).

Cell cultures were successfully established from both large and small airway brushings with a similar success rate (83.8 ± 4.7% and 79.0 ± 5.2%; p = 0.49 respectively). Established cultures reached confluence within a median 21 days (range 13 - 57 days) and maintained a polygonal, cobblestone appearance, typical of epithelial cells. No major morphological variations were observed between LAEC and SAEC over the life of the culture (Fig 3). Immunocytochemistry conducted on cells from passage 0 (p0) and passage 2 (p2) with epithelial, and mesenchymal markers confirmed the preservation of epithelial lineage of the cells.

Culture fates are presented in Table 2. Successful establishment was limited predominantly by superinfection by organisms colonizing the transplanted organ (2 patients, *A. fumigatus *and *S. aureus*) and low cell yield. The latter problem was confined to SAEC - failure of five of the cultures could be attributed to low cell yield and they were all from small airway brushings. The mean cell yield for the five failed cultures was 0.326 ± 0.055 × 10^6 ^cells, which is significantly lower than the cell yield for successful cultures (1.071 ± 0.070 × 10^6 ^cells; p < 0.01). The presence of BOS significantly compromised culture success for SAEC (odds ratio (95%CI) 0.067 (0.01-0.40)) but not for LAEC (0.3 (0.05-1.9)). Since the cell yield was not different for BOS SAEC, poor culture establishment did not appear to be related to low starting cell numbers.

**Table 2:** Fate of cultures established from small airway (SAEC) and large airway (LAEC) brushings

				Successful Culture Bronchoscopy Number									
**Patient No.**	**Sex**	**Age**	**BOS grade**	**1**		**2**		**3**		**4**		**5**	

				**SAEC**	**LAEC**	**SAEC**	**LAEC**	**SAEC**	**LAEC**	**SAEC**	**LAEC**	**SAEC**	**LAEC**
**1**	f	18	0	Y	Y	N^γ^	N^γ^	Y	Y	-	-	-	-
**2**	m	50	0	N^δ^	Y	-	-	-	-	-	-	-	-
**3**	m	46	1	N^δ^	Y	-	-	-	-	-	-	-	-
**4**	f	55	0	Y	N^α^	Y	Y	-	-	-	-	-	-
**5**	m	40	0	Y	Y	Y	Y	Y	Y	Y	Y	Y	Y
**6**	m	61	0	N^γ^	N^γ^	Y	Y	-	-	-	-	-	-
**7**	m	60	1	N^γ^	N^γ^	N^δ^	Y	Y	Y	-	-	-	-
**8**	f	25	0	Y	Y	N^δ^	Y	N^δ^	Y	Y	Y	-	-
**9**	m	58	0	Y	Y	Y	Y	-	-	-	-	-	-
**10**	f	55	0	Y	Y	Y	Y	Y	Y	-	-	-	-
**11**	f	58	3	N^γ^	N^γ^	-	-	-	-	-	-	-	-
**12**	m	59	0	Y	Y	Y	Y	Y	Y	-	-	-	-
**13**	f	55	0	N^γ^	N^γ^	Y	Y	Y	Y	-	-	-	-
**14**	m	44	0	N^γ^	Y	Y	Y	Y	Y	Y	Y	Y	Y
**15**	m	40	2	Y	Y	N^β^	N^β^	-	-	-	-	-	-
**16**	m	51	0	Y	Y	Y	Y	N^γ^	N^γ^	Y	Y	-	-
**17**	f	58	0	Y	Y	-	-	-	-	-	-	-	-
**18**	m	51	0	Y	Y	Y	Y	Y	Y	Y	Y	Y	Y
**19**	m	64	0	Y	Y	Y	N^γ^	-	-	-	-	-	-
**20**	m	49	0	Y	Y	-	-	-	-	-	-	-	-
**21**	f	61	0	Y	Y	Y	Y	-	-	-	-	-	-
**22**	f	54	0	Y	Y	Y	Y	-	-	-	-	-	-
**23**	m	59	0	Y	Y	Y	Y	-	-	-	-	-	-
**24**	m	54	0	Y	Y	-	-	-	-	-	-	-	-
**25**	f	25	0	Y	Y	-	-	-	-	-	-	-	-
**26**	f	27	0	Y	Y	-	-	-	-	-	-	-	-

*Y - Successful Culture

*N^α^-Unsuccessful culture due to superinfection with *A. fumigatus*

*N^β ^- Unsuccessful culture due to superinfection with *S. aureus*

*N^γ ^- Unsuccessful culture due to other causes

*N^δ ^= Unsuccessful culture due to low cell yield

### Epithelial lineage verification

**Figure 4:**
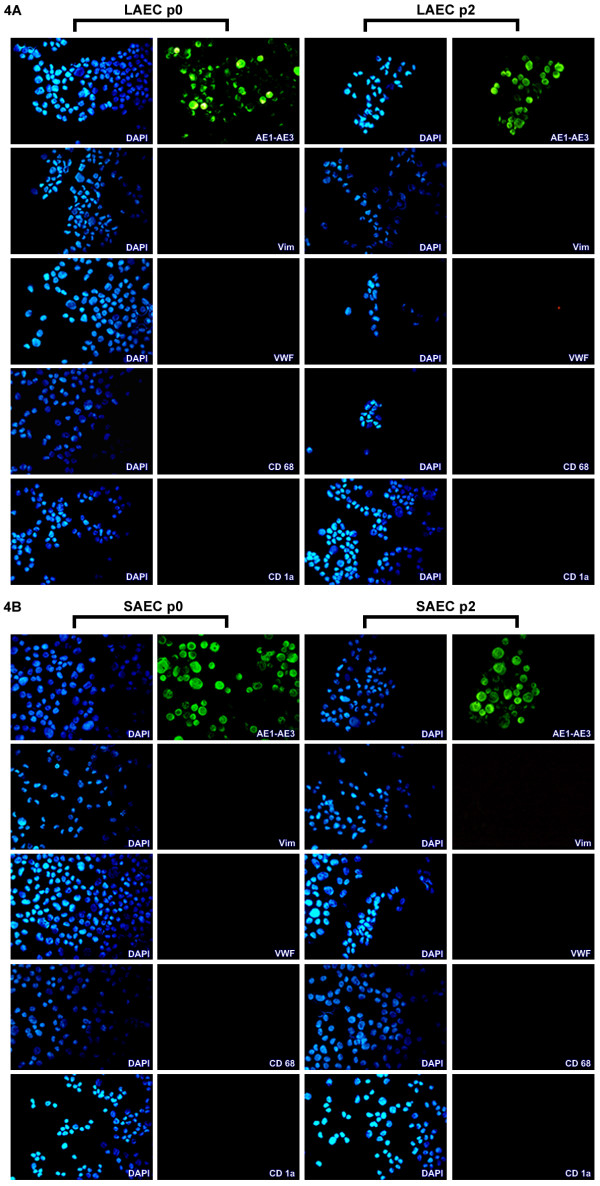
**Characterisation of established epithelial cell cultures**. Cytospins obtained from cells cultured from large airway bronchial brushing (LAEC; Fig. 4A) and small airway brushings (SAEC; Fig. 4B) from representative lung allograft samples (at p0 and p2) were incubated with primary antibodies specific for mesenchymal (Vimentin (Vim)), endothelial (von Willebrand factor (VWF)), macrophage (CD68), dendritic (CD1a), and epithelial lineages (AE1-AE3) for 24 hours at 4°C followed by fluorescently conjugated secondary antibodies for a similar period. The slides were counterstained with 4', 6-diamidino-2-phenylindole (DAPI), which illuminates cell nuclear material (blue). Results confirmed that established cultures were not contaminated by any other cell types since and were considered pure epithelial cultures by the sole expression of all cells with the epithelial lineage marker AE1-AE3 (magnification 400×).

Morphological analysis of established cultures over repetitive passage showed that the typical cobblestone morphology indicative of epithelial cells was maintained over culture duration. Epithelial lineage was further verified at each passage via immunocytochemical staining. Cultured SAEC and LAEC stained intensely and exclusively for the epithelial specific marker, AE1-AE3 (cytokeratin) at both p0 and p2. No expression was observed for mesenchymal (Vimentin), macrophage (CD68), dendritic (CD1a) or endothelial (Von Willebrand Factor) lineage markers at either passage (Fig. 4).

### Small airway epithelial lineage verification

**Figure 5:**
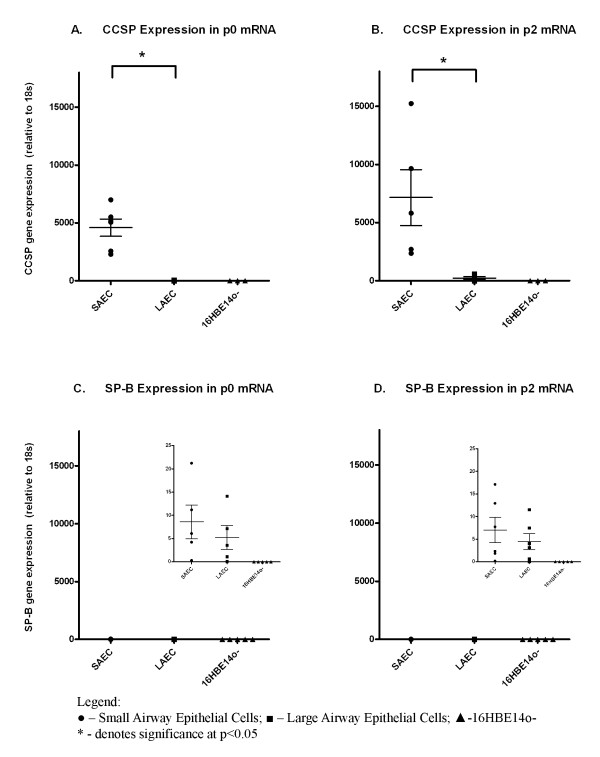
**Lineage verification of small airway epithelial cells**. **A & B**; Gene expression of CCSP (CC-10) in small airway (SAEC) cell cultures, large airway cell cultures (LAEC) at p0 and p2 as well as large airway epithelial cell line 16HBE14o^- ^cells as compared to the housekeeping gene 18 s. The expression of CCSP in SAEC was significantly higher than in LAEC at both p0 (p = 0.0001) and p2 (p = 0.0113) and no expression was observed in 16HBE14o^-^. **C & D**: Gene expression of Surfactant Protein-B (SP-B) in small airway (SAEC) cell cultures, large airway cell cultures (LAEC) at p0 and p2 as well as large airway epithelial cell line 16HBE14o^- ^cells as compared to the housekeeping gene 18 s. The expression of SP-B in small airways was seen to be markedly lower than CCSP. Furthermore, the expression of SP-B in SAEC was not significantly different than in LAEC at both p0 (p = 0.4660) and p2 (p = 0.4607) (inset) and no expression was observed in 16HBE14o^-^.

Lineage verification of established cultures was then assessed using known and suggested markers of small airway epithelium [11,12]. Here, we assessed small airway gene expression of CCSP and SP-B using qPCR on RNA extracted from cultures at p0 and p2. CCSP was exclusively expressed in SAEC (4592 ± 743.4 fold normalized to 18 s at p0; 7148 ± 5385 fold normalized to 18 s at p2) compared to LAEC (11.56 ± 9.113 fold normalized to 18 s at p0, p = 0.0001; 235 ± 275.6 normalized to 18 s at p2, p = 0.0113). CCSP was not expressed in a LAEC immortalized cell line (16HBE14o^- ^cells (Fig. 5A &5B)). To exclude an alveolar source for the small airway brush cellular material, SP-B gene expression was also assessed in large and small airway cell cultures at p0 and p2. SP-B was expressed only at low levels in both SAEC and LAEC at p0 and p2. There was no difference between SAEC and LAEC SP-B expression (p = NS at both p0 & p2). SP-B was not expressed by 16HBE14o^- ^cells (Fig. 5C &5D).

## Discussion

We have developed a method for successfully collecting and establishing expandable primary cultures from human SAEC obtained bronchoscopically. The method was well tolerated and easy to perform. The cell yield using this collection method was lower in small airway brushings than the large airway brushings however this did not significantly compromise the culture establishment rate. Both LAEC and SAEC maintained their lineage over passage.

The inability to establish a suitable *in vitro *model using primary human cells has been a major impediment to research into post-transplant chronic allograft dysfunction. The most relevant *in vitro *work has been conducted on human LAEC despite BOS being a disease of small airways [7,13]. It is highly probable that *in vitro *work in LAEC can not be neatly extrapolated to SAEC. The described methods will facilitate the development of more relevant *in vitro *models not only for OB, but also for other diseases with small airway pathology. In the case of transplantation for instance, OB is the result of a range of alloreactive, infective and non-specific insults and recent evidence suggests that transforming growth factor β (TGF-β_1_) driven epithelial mesenchymal transition (EMT) is the final common pathway to airway obstruction and fibrosis[14]. Using the model described herein, EMT can be induced by TGF-β_1 _*in vitro*, with the ability to assess candidate compounds for therapeutic efficacy.

Several investigators have successfully established LAEC cultures from bronchial brushings, which have been used to study a wide range of diseases including OB [7,13], asthma [8], cystic fibrosis [15] and COPD [16]. The present study has extended these methods to SAEC. The LAEC collection and extraction methods described here are very similar to those reported by Forrest *et. al. *[17]. The cell yield in this case was lower than that reported (~4.1 × 10^6 ^cells) but the number of brushings conducted was also lower in comparison (2-3 vs. 4-6)[17]. However, when compared to cell yields from non-transplant [18] or paediatric patients [8], yields are much lower. Reasons behind the reduced yield are unclear but may be specific to the post-transplant state or related to medication. Although large numbers of bronchial epithelial cells have been collected from sources such as surgically resected lung [19], explanted lung [20] and cadavers [21], sample availability severely limits the utility of this approach in high turnover laboratory projects. Conversely, using brushings from lungs from living patients not only allows the collection of a much higher numbers of samples, but also facilitates longitudinal analyses and the more rapid translation of *in vitro *studies to clinical practice.

As with LAEC, SAEC from whole lung or resection tissue have been successfully cultured using similar methodologies [20,21]. SAEC have also been obtained using an ultrathin fibrescope [22-24] or unguided bronchial brushings [12]. However, only one laboratory has successfully cultured human SAEC from bronchial brushings [25], derived from smokers, COPD patients and controls using an ultrathin fibrescope. These SAEC were directly cultured in 48 well plates and harvested for ICC. Although epithelial lineage was verified, no data was reported confirming small airway lineage [25], and the utility of this *in vitro *model was limited since cultures were not expanded through repeated passage. Expanded cultures facilitate varied experiments and the acquisition of multifaceted data including cellular morphology, gene and protein expression, and soluble protein expression in culture supernatant.

The culture establishment rate was higher than that reported by similar studies [17], but was still compromised by contamination with passenger organisms, insufficient cell yield and BOS. Bacterial and fungal contamination occurred despite the inclusion of antibiotic (gentamycin, penicillin and streptamycin) and anti-fungal (amphotericin B) agents. Attempts at increasing the doses of these agents in culture media were unsuccessful due to cytotoxicity (data not shown). Unfortunately endemic infection with opportunistic pathogens is common in this patient group and has been previously noted to compromise large airway cell culture [13,17]. In this study, we interestingly observed that establishing cell cultures from small airway (but not large airway) brushings of BOS patients was more difficult. Forrest *et al *did not report the effect of BOS grade on culture establishment [17], and we can find no other literature on the topic. Further investigation revealed revealed that the inability to establish cultures from BOS affected small airways was independent of both cell yield and the presence of passenger organisms (Table 2). Collectively, our results suggest that identifying the reasons for poor culture establishment may in fact provide insight into BOS pathogenesis. In this regard, our laboratory is currently investigating epithelial cell phenotype and function in BOS, the progenitor capacity and proliferative potential of these cells, as well as their tendency to undergo programmed cell death or senescence.

## Conclusion

In conclusion, we have developed methodology for successfully collecting and culturing SAEC from humans during bronchoscopy. The techniques employed utilise commonly available equipment, facilitating easy and consistent sample collection. Given the importance of the small airways in a number of pulmonary diseases, including OB, the methods established here could facilitate several avenues of respiratory research. The present authors are using SAEC from transplanted lungs to develop an *in vitro *model for OB, however the sampling techniques described could be used to develop small airway submerged or air liquid interface culture models for diseases such as asthma, cystic fibrosis and COPD.

## Competing interests

The authors declare that they have no competing interests.

## Authors' contributions

BB collected and processed the majority of the brushings, established cell cultures as well as conducted lineage verification by immunohistochemistry and qPCR. BB also drafted the manuscript and performed all statistical analysis. AK optimised and established the protocols for cell culture, assisted with the design of the study, assisted in sample collection and processing, critically revised the manuscript and assisted with the statistical analyses. MM performed the bronchial brushings of patients during bronchoscopy. ES assisted with cell culture establishment and expansion as well as initial sample processing. SS was involved in the design and coordination of the study. DC initially conceived the study and was responsible for its design, analysis of results and assisted with drafting the manuscript. All authors read and approved the manuscript.
